# Functional status and annual hospitalization in multimorbid and non-multimorbid older adults: a cross-sectional study in Southern China

**DOI:** 10.1186/s12955-018-0864-4

**Published:** 2018-02-13

**Authors:** Xiao-Xiao Wang, Zhao-Bin Chen, Xu-Jia Chen, Ling-Ling Huang, Xiao-Yue Song, Xiao Wu, Li-Ying Fu, Pei-Xi Wang

**Affiliations:** 1 Institute of Public Health, School of Nursing and Health, Henan University, Kaifeng, 475004 China; 2 West China School of Public Health, Sichuan University, Chengdu, Sichuan 610041 China; 3 Shenzhen Nanshan Center for Disease Control and Prevention, Shenzhen, 518054 China; 4 Community health service management center, Luohu hospital group, Shenzhen, 518007 China; 5 The Nursing College Of Zhengzhou University, Zhengzhou, 450001 China; 6 Department of Preventive Medicine, School of Public Health, Guangzhou Medical University, Guangzhou, 510182 China

**Keywords:** Hospitalization, Functional status, Multimorbidity, Older adults, Cross-sectional study, China

## Abstract

**Background:**

Hospitalization over the last one year, an indicator of health service utilization, is an important and costly resource in older adult care. However, data on the relationship between functional status and annual hospitalization among older Chinese people are sparse, particularly for those with and without multimorbidity. In this study,we aimed to examine the association between functional status and annual hospitalization among community-dwelling older adults in Southern China, and to explore the independent contributions of socio-demographic variables, lifestyle and health-related factors and functional status to hospitalization in multimorbid and non-multimorbid groups.

**Methods:**

This cross-sectional, community-based survey, studied 2603 older adults aged 60 years and above. Functional status was assessed by Functional Independence Measure (FIM). The outcome variable was any hospitalization over the last one year (annual hospitalization). Clustered logistic regression was used to analyze the independent contributions of FIM domains to annual hospitalization.

**Results:**

Only in the multimorbid group, did the risk of annual hospitalization decrease significantly with increasing FIM score in walk domain (adjusted OR = 0.80 per SD increase, 95% CI = 0.70–0.91, *P* = 0.001) and its independent contribution accounted for 24.62%, more than that of socio-demographic variables (18.46%). However, among individuals without multimorbidity, there were no significant associations between FIM domains and annual hospitalization; thus, no independent contribution to the risk of hospitalization was observed.

**Conclusions:**

There exist some degree of correlation between functional status and annual hospitalization among older adults in Southern China, which might be due to the presence of multimorbidity with advanced age.

## Background

The population of China is aging much faster than those of other high-income or low- and middle-income countries, with the proportion of the population aged 60 years and over is predicted to increase from 12.4% in 2010 to 28% in 2040 [1], which has become a major concern. Aging is strongly associated with increased multiple chronic conditions [2, 3] and functional decline [4], which in turn leads to substantial increases in health resources usage and costs [5–8]. Hospitalization in the last year, as a part of health service utilization, is an important and costly resource in older adult care. Numerous studies have examined the determinants of hospitalization, including, age, gender [9], multiple chronic conditions [5, 10, 11], and activities of daily living (ADL) limitations [12]. Several studies also have found that hospitalization, especially if repeated and prolonged, might be associated with negative consequences including an increased risk of falls [13], worsening or irreversible functional decline [13–15], and death [16]. To reduce hospitalization and its associated adverse outcomes, it is important to identify and understand the risk factors of hospitalization in older adults.

Among community-dwelling individuals, the ability to take care of themselves or survive in the community is an important aspect of quality of life. The Functional Independence Measure is one of the instruments to measure functional status [17]. Generally, loss of function is related to advancing age. Many of the very old lose their ability to live independently owing to limited mobility, frailty or other physical or mental health problems, which consequently require additional health services, particularly unplanned hospitalization. Multimorbidity (≥2 chronic diseases) is common in older adults. There have been many studies exploring the correlations of multimorbidity, functional status [18–20] and hospitalization [21, 22]. A population-based study in Brazil showed that disability was strongly associated with hospitalization, yet it also suggested that functional health dimensions have not oriented health services, still largely conditioned on the presence of diseases [23]. Moreover, Mor V and colleagues [24] documented the link between functional decline and increased hospital use. However, they also indicated that the “true” causes of hospitalization are less clear as functional status is likely affected by multiple chronic diseases and age-related processes. Unfortunately, the issue was simply mentioned and failed to be deeply studied. Therefore, exploring the association of functional status and hospitalization based on multimorbidity stratification is important and necessary.

With the rapid aging of population in China, the health of older adults has been deeply concerned. Thus, in this study, we selected the community-dwelling older adults in Southern China and examined the association between functional status and annual hospitalization, to explore the independent contributions of socio-demographic variables, lifestyle and health-related factors and functional status to hospitalization in multimorbid and non-multimorbid groups.

## Methods

### Study design and populations

The data were based on a cross-sectional community health diagnosis survey in a district of Shenzhen City, Guangdong Province, China, 2015. The survey samples were selected using a multistage sampling method of family members drawn from 5 % of the total populations in this region. The primary sampling units were street communities, second-stage sampling units were communities and the stratification was according to the economic level. All information were obtained by face to face interviews in residents’ homes. The sample size was decided according to the hospitalization rate and calculated by the formula of sample size for rate. Supposing that the hospitalization rate was 15% according to the results of pre-survey, the sample size of this study was sufficient based on the community health diagnosis survey. This study selected available information from participants over the age of 60 years. Of 2919 participants, 316 provided incomplete questionnaire data and the response rate was 89.2%. Finally, data from a total of 2603 older adults were included in our final analysis.

### Measurements and instruments

#### Participant characteristics

We firstly conducted a literature search to identify the potential factors related to annual hospitalization in older adults. This search yielded the following variables: age, gender, marital status, employment status, smoking, physical activities, body mass index, chronic conditions, functional status [5–7, 9, 10, 23–26]. Therefore the variables in this study analysis included socio-demographic characteristics (age, gender, marital status, etc), lifestyle and health-related factors (smoking, physical activities, etc), functional status and hospitalization over the last one year. Current smoking status was defined as smoking one or more cigarettes per day for at least six months. Drinking was defined as the consumption of at least thirty-seven milliliter of alcohol per week. Exercise was assessed by the responses to the question: “How many times do you exercise every week? (more than three times/week, one to two times/week and no exercise)”. The BMI was calculated as weight (kg) divided by the square of height (m^2^). Information on annual hospitalization was obtained by the responses to the question: “Have you been hospitalized in the last year? (yes/no)” .

### Assessment of functional status

Functional status was assessed using the Functional Independence Measure (FIM). The FIM score was defined as the level of assistance required for an individual to perform ADL, which indicated the burden of caring for them [27]. The tool includes two parts (motor function and cognition function), six domains and 18 items. Among six domains, self-care ability has six items, including eating, grooming, bathing, upper body dressing, lower body dressing and toileting. Sphincter control includes bladder management and bowel management. Transfer includes bed to chair transfer, toilet transfer and shower transfer. Walk domain includes locomotion and stairs. Communication includes cognitive comprehension and expression. Social cognition includes social interaction, problem solving and memory. Each item is scored from 1 to 7 based on the level of independence, where 1 represents total assistance (patient can perform less than 25% of the task or requires more than one person to assist) and 7 indicates complete independence [28]. Possible scores range from 18 to 126, with lower scores indicating less functional independence. Functional status can be divided into functional dependence (< 108 scores) and functional independence (108–126 scores). As we know, the 18-item FIM instrument has been reported to be reliable and well validated [29–31] and can be widely used in China [32].

### Multimorbidity

Multimorbidity was defined as the presence of two or more chronic diseases in an individual [33]. The number of chronic diseases was self-reported based on the responses to the question, “Has a doctor ever diagnosed that you had…(yes/no)” [34]. The chronic diseases investigated in this study included: hypertension, chronic pain, diabetes mellitus, hyperlipidemia, bone diseases, chronic gastrointestinal diseases, heart disease, gout, peripheral vascular disease, chronic kidney disease, spleen and gallbladder diseases, pulmonary disease, stroke, cancer, multiple sclerosis, dementia and mental disorder.

### Statistical analysis

Means and standard deviations (SD) were presented for continuous variables, while frequency and percentage were used for categorical variables. The main dichotomous outcome variable was annual hospitalization. To evaluate the differences in the distributions of multimorbidity by continuous or categorical variables, we used *t*-tests or chi-square tests, as appropriate. Logistic regression was employed to calculate the odds ratios (ORs) and 95% confidence intervals (95% CIs) for the associations between FIM six domains and annual hospitalization. During the regression analysis, continuous variables (including age, BMI, number of chronic diseases and FIM domains scores) were standardized in order to make the data comparable.

Subsequently, clustered logistic regression [35, 36] was used to explore the impacts of socio-demographic characteristics, lifestyle and health-related factors and FIM domains (three clusters based on the nature of the study variables) on annual hospitalization. Multidirectional associations may exist among three clusters and the dependent variable. To be specific, cluster 1 may impact cluster 2, cluster 3 and the outcome variable. Likewise, cluster 2 may affect cluster 3 and the dependent variable, while cluster 3 may impact the outcome variable. Consequently, simultaneous consideration of variables from the clusters in a free multiple regression model (i.e. a free forward stepwise logistic regression model) might bring about confounded inference. Thus, clustered logistic regression [35] was adopted to analyze whether the addition of FIM variables to the models including socio-demographic and lifestyle and health-related variables could significantly increase the explanatory power of the risk adjustment models. The final regression model was determined in three steps: (1) A forward stepwise regression of annual hospitalization for the cluster 1 variables; (2) A forward stepwise regression for the cluster 2 variables with the equation derived from step 1 as a fixed part of the new regression model; (3) A forward stepwise regression for the cluster 3 variables with the equation derived from step 2 as a fixed part of the new regression model. Variables include and exclude criteria for the stepwise regression models were *P* values of 0.05 and 0.10, respectively.

The independent effect of each cluster was assessed by the corresponding R^2^ value. The independent contribution share of each cluster was calculated as individual R^2^ change / total R^2^ change in the final model × 100%. The R^2^ in logistic regression models was the Nagelkerke “pseudo” R^2^, similar to the classical R^2^ in linear regression models for data interpretation [36].

All statistical analyses were conducted using the Statistical Package for the Social Sciences (SPSS), version 17.0 (SPSS Inc., Chicago, IL, USA). Two-tailed *P* values below 0.05 were considered statistically significant.

## Results

Among 2603 older adults (aged 60 years and above), with an average age of (69.28 ± 7.59) years, the majority were women (57.90%), married (76.80%), and unemployed (95.00%). More than half of the subjects had an education level of primary school or lower. Regarding lifestyle and health-related factors, most did not smoke (86.50%), drink (85.50%), and exercised more than three times per week (60.30%). The average BMI was (23.90 ± 3.87) kg/m^2^. The most frequent chronic diseases included hypertension, chronic pain, diabetes mellitus and so on. About 45.06% of older adults reported multimorbidity. The FIM scores in six domains varied from 19.82 ± 3.02 to 41.14 ± 4.02. Among the six FIM domains, walk scored the lowest, while self-care ability scored the highest. The prevalence of annual hospitalization was 10.50% and was significantly higher in the multimorbid group (*P* < 0.001). More details of participants’ characteristics among participants with and without multimorbidity are shown in Tables 1 and 2.

**Table 1:** Variables and assignments

Variable	Assignment
Cluster1: socio-demographic factors	
Age (years)	
Gender	1 = Male, 2 = Female
Marital status	1 = Married, 2 = Single
Education level	1 = Primary school or lower, 2 = Middle school, 3 = High school or above
Employment status	1 = Employed, 2 = Unemployed
Individual economic condition	1 = Good, 2 = Not good
Medical insurance	1 = Yes, 2 = No
Cluster2: lifestyle and health-related factors	
Smoking	1 = Yes, 2 = No
Drinking	1 = Yes, 2 = No
Physical exercise	1 = Over 3 times/week, 2 = 1–2 times/week, 3 = Take no exercise
Body mass index	
Sleep status	1 = Good, 2 = Not good
History of chronic diseases ^a^	1 = Yes, 2 = No
Absolute number of chronic diseases	
Cluster3: FIM domains	
Self-care ability	
Sphincter control	
Transfer	
Walk	
Communication	
Social cognition	
Outcome	
Hospitalization in the last year	0 = No, 1 = Yes

*FIM* Functional Independence Measure

Single: unmarried, divorced and widowed. Multimorbidity was defined as the presence of two or more chronic diseases in an individual

^a^History of chronic diseases, including hypertension, chronic pain, diabetes mellitus, hyperlipidemia, bone diseases, chronic gastrointestinal diseases, heart disease, gout, peripheral vascular disease, chronic kidney disease, spleen and gallbladder diseases, pulmonary disease, stroke, cancer, multiple sclerosis, dementia, and mental disorder

**Table 2:** Study participant characteristics stratified by multimorbidity

Variable	Multimorbid (*n* = 1173)	Non-multimorbid (*n* = 1430)	*P* value
*Socio-demographic factors*			
Age (years)	70.08 (7.7)	68.62 (7.4)	< 0.001^a^
Gender (male)	448 (38.2)	647 (45.2)	< 0.001^b^
Marital status (married)	840 (71.6)	1158 (81.0)	< 0.001^b^
Education level			0.044^b^
Primary school or lower	587 (50.0)	756 (52.9)	
Middle school	249 (21.2)	325 (22.7)	
High school or above	337 (28.7)	349 (24.4)	
Employment status (yes)	38 (3.2)	92 (6.4)	< 0.001^b^
Economic condition (good)	627 (53.5)	796 (55.7)	0.029^b^
Medical insurance (yes)	1158 (98.7)	1415 (99.0)	0.058^b^
*Lifestyle and health-related factors*			
Smoking (yes)	143 (12.2)	208 (14.5)	0.080^b^
Drinking (yes)	171 (14.6)	206 (14.4)	0.901^b^
Exercise			0.006^b^
Over 3 times/week	716 (61.0)	854 (59.7)	
1–2 times/week	302 (25.7)	325 (22.7)	
Take no exercise	155 (13.2)	251 (17.6)	
Body mass index score	24.19 (4.1)	23.66 (3.6)	0.001^a^
Sleep status (good)	579 (49.4)	894 (62.5)	< 0.001^b^
Hypertension (yes)	808 (68.9)	360 (25.3)	< 0.001^b^
Chronic pain (yes)	574 (48.9)	128 (9.0)	< 0.001^b^
Diabetes mellitus (yes)	352 (30.0)	88 (6.2)	< 0.001^b^
Hyperlipidemia (yes)	330 (28.1)	30 (2.1)	< 0.001^b^
Bone diseases (yes)	289 (24.6)	40 (2.8)	< 0.001^b^
Chronic gastrointestinal diseases (yes)	261 (22.3)	45 (3.1)	< 0.001^b^
Heart disease (yes)	244 (20.8)	26 (1.8)	< 0.001^b^
Gout (yes)	167 (14.2)	15 (1.0)	< 0.001^b^
Peripheral vascular disease (yes)	138 (11.8)	12 (0.8)	< 0.001^b^
Chronic kidney disease (yes)	104 (8.9)	10 (0.7)	< 0.001^b^
Spleen and gallbladder diseases (yes)	94 (8.0)	10 (0.7)	< 0.001^b^
Pulmonary disease (yes)	89 (7.6)	10 (0.7)	< 0.001^b^
Stroke (yes)	71 (6.1)	6 (0.4)	< 0.001^b^
Cancer (yes)	23 (2.0)	4 (0.3)	< 0.001^b^
Multiple sclerosis (yes)	17 (1.4)	2 (0.1)	< 0.001^b^
Dementia (yes)	16 (1.4)	1 (0.1)	< 0.001^b^
Mental disorder (yes)	4 (0.3)	2 (0.1)	0.419^b^
Number of chronic diseases	3.05 (1.4)	0.55 (0.5)	< 0.001^a^
*FIM domains scores*			
Self-care ability	40.87 (4.7)	41.36 (3.4)	< 0.001^a^
Sphincter control	13.63 (1.6)	13.79 (1.2)	< 0.001^a^
Transfer	19.55 (3.3)	20.03 (2.7)	< 0.001^a^
Walk	12.62 (2.5)	13.12 (2.1)	< 0.001^a^
Communication	13.00 (2.3)	13.29 (1.8)	< 0.001^a^
Social cognition	18.58 (3.3)	19.29 (2.7)	< 0.001^a^
*Outcome*			
Annual hospitalization			< 0.001^b^
No	986 (84.1)	1344 (94.0)	
Yes	187 (15.9)	86 (6.0)	

*FIM* Functional independence measure

Data presented are mean (SD) or n (%); Multimorbidity defined as the presence of two or more of chronic diseases in an individual

^a^Based on *t*-test

^b^Based on chi-square test

Table 3 shows the associations between FIM domains and annual hospitalization among older adults stratified by multimorbidity. In the multimorbid group, higher FIM scores in walk and social cognition domains were significantly associated with lower odds of hospitalization, namely, the risk of hospitalization increased significantly with decreasing FIM scores in walk and social cognition domains. For non-multimorbid subjects, a lower FIM score in walk domain was associated with increased risk of hospitalization.

**Table 3:** Associations between FIM domains and annual hospitalization in multimorbid (*n* = 1173) and non-multimorbid (*n* = 1430) older adults^a^

Variable	Annual Hospitalization					
	OR ^b^			95% CI		
	Multimorbid	Non-multimorbid	Total	Multimorbid	Non-multimorbid	Total
Self-care ability	1.01	1.03	1.05	0.78–1.29	0.61–1.72	0.84–1.31
Sphincter control	1.03	1.27	1.04	0.82–1.28	0.75–2.16	0.85–1.27
Transfer	0.98	0.93	0.96	0.78–1.22	0.70–1.24	0.81–1.14
Walk	0.79^*^	0.72^*^	0.75^*^	0.66-0.95	0.56-0.94	0.64–0.87
Communication	1.28	0.93	1.19	1.04–1.58	0.69–1.24	1.00–1.41
Social cognition	0.78^*^	1.06	0.81^*^	0.63-0.97	0.75-1.49	0.68–0.97

*FIM* Functional Independence Measure

^*^P < 0.05

^a^The six FIM domains were included as predictor variables for annual hospitalization in a multivariable regression model without adjusting for other variables

^b^Odds ratio per SD increase in a predictor variable

**Fig. 1:**
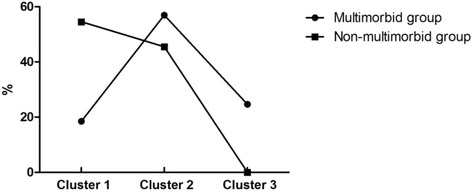
The independent contributions of three clusters to annual hospitalization between participates with and without multimorbidity

Several clustered logistic regression models are shown in Table 4 and Table 5*.* The independent contributions of three clusters to annual hospitalization among older adults with and without multimorbidity are illustrated in Fig. 1. Among socio-demographic variables (cluster 1), in the multimorbid group, only gender (male) was significantly associated with hospitalization (*P* = 0.004), while age had a significant association with annual hospitalization in non-multimorbid participants (*P* < 0.001). The independent contributions from social demographic variables with multimorbidity stratification were 18.46% and 54.55%, respectively. In cluster 2 (lifestyle and health-related factors), among participants with multimorbidity, the number of chronic diseases was significantly associated with annual hospitalization, while in non-multimorbid group, diabetes mellitus, peripheral vascular disease and heart disease are the significant risk factors for annual hospitalization. The independent contributions of the second cluster in older adults with and without multimorbidity were 56.92% and 45.45%, respectively. In the third cluster, the risk of annual hospitalization decreased significantly with increasing FIM score in walk domain (adjusted OR = 0.80 per SD increase, 95% CI = 0.70–0.91, *P* = 0.001) only in multimorbid group and its independent contribution to hospitalization was 24.62%. However, for those older adults without multimorbidity, there were no associations between all FIM domains and dependent variable; consequently, no independent contribution of the third cluster was observed.

**Table 4:** Clustered logistic regression models explaining hospitalization in the last year by socio-demographic characteristics, lifestyle and health-related factors, and FIM domains among patients with multimorbidity (*n*=1173)

Variable^a^	OR ^b^	95% CI	*P* value	Nagelkerke R^2c^	Independent contribution ^d^ (%)
Model 1					
Gender (male)	1.59	1.16–2.17	0.004		
Total				0.012	18.46
Model 2					
Gender (male)	1.60	1.16–2.20	0.004		
Number of chronic diseases	1.52	1.30–1.78	< 0.001		
Total				0.049	56.92
Model 3					
Gender (male)	1.63	1.18–2.24	0.003		
Number of chronic diseases	1.45	1.24–1.71	< 0.001		
Walk	0.80	0.70–0.91	0.001		
Total				0.065	24.62

^a^Only variables with *P* < 0.05 were included in the model

^b^For age, body mass index, number of chronic diseases, and functional independence domains scores, the odd ratios per SD increase are shown

^c^Nagelkerke R^2^ is the variance of the dependent variable (hospitalization in the last year) explained by all independent variables included in the regression model

^d^The independent contribution of each cluster of predictors to the variation in hospitalization in the last year calculated as individual corresponding R^2^ change/total R^2^ change in the final model × 100%

**Table 5:** Clustered logistic regression models explaining hospitalization in the last year by socio-demographic characteristics, lifestyle and health-related factors, and FIM domains among patients without multimorbidity (*n* = 1430)

Variable^a^	OR^b^	95% CI	*P* value	Nagelkerke R^2c^	Independent contribution^d^ (%)
Model 1					
Age	1.47	1.20–1.79	< 0.001		
Total				0.048	54.55
Model 2					
Age	1.51	1.23–1.85	< 0.001		
Diabetes mellitus	2.61	1.27–5.35	0.009		
Peripheral vascular disease	8.75	2.22–34.47	0.002		
Heart disease	3.93	1.42–10.92	0.009		
Total				0.088	45.45
Model 3					
Age	1.51	1.23–1.85	< 0.001		
Diabetes mellitus	2.61	1.27–5.35	0.009		
Peripheral vascular disease	8.75	2.22–34.47	0.002		
Heart disease	3.93	1.42–10.92	0.009		
Total				0.088	0

^a^Only variables with *P* < 0.05 were included in the model

^b^For age, body mass index, number of chronic diseases, and functional independence domains scores, the odd ratios per SD increase are shown

^c^Nagelkerke R^2^ is the variance of the dependent variable (hospitalization in the last year) explained by all independent variables included in the regression model

^d^The independent contribution of each cluster of predictors to the variation in hospitalization in the last year calculated as individual corresponding R^2^ change/total R^2^ change in the final model × 100%

Additionally, we also performed a chi-square test to analyze the association between FIM category and annual hospitalization among older adults with and without multimorbidity. The results found that functional status was significantly associated with annual hospitalization among participants with multimorbidity, (*P* = 0.003), whereas there was no association in non-multimorbid group (*P* > 0.05). The multimorbid participants with functional dependence had a higher rate of hospitalization (25.2% VS 14.8%).

## Discussion

### Main findings

This study was to explore the association of functional status and hospitalization in multimorbid and non-multimorbid older Chinese adults. The results showed that the risk of annual hospitalization increased with lower scores in certain FIM domains in multimorbid group, and the independent contribution of FIM domains to hospitalization was larger than that of socio-demographic characteristics. Whereas no contribution of FIM domains was observed among non-multimorbid older adults. These findings suggested that the association of functional status on annual hospitalization might be likely due to the presence of multimorbidity with advanced age.

### Comparing with previous studies

Our study showed that walk domain of FIM was significantly associated with hospitalization in multimorbid older adults. A higher walk score was associated with lower odds of annual hospitalization after controlling for socio-demographic characteristics and lifestyle and health-related factors. The results were consistent with a US Renal Data System special study [37] that reported that low walk speed was associated with an increased likelihood of ADL difficulty and hospitalization in the geriatric population. Another survey also found that a single disability variable (use of cane, walker, or wheelchair) was a predictor of hospitalization [10]. Many studies have used different methods to measure functional status to demonstrate the relationship between functional status and hospital admissions or readmissions [23, 38, 39]. A population-based study including 1624 elderly patients (≥60 years) found that both ADL and IADL were significantly associated with hospitalization [23]. There was another cohort study to examine the independent association of activity limitation stages with risk of hospitalization within a year in elderly Medicare beneficiaries, showing that the adjusted risk of first hospitalization increased with higher activity limitation stages [38]. Older people’s poor functional independence is not only an important indicator of poor health but also might exacerbate the severity of underlying health problems, resulting in increased hospitalization. Additionally, poor functional independence is associated with higher frequency of accidents, increased risk of falls [40], and multiple chronic diseases. However, unfortunately, most studies neglected the important effect of co-morbidity, which is a common risk for disability and hospitalization. In our study, the important findings were that the walk domain score was significantly associated with hospitalization only in the multimorbid group, whereas no contribution of FIM domains was observed among older adults without multimorbidity. These findings suggest that the increased risk of hospitalization may be conditioned on the presence of multiple chronic diseases, similar to the findings of Fialho [23]. Also, Mor et al. [24] reported that chronic illness is a robust predictor of all future outcome states, even more so than age and nearly as much as function. There was no denying that, when compared to those without multimorbidity, multimorbid individuals mostly have older age and poorer functional ability. Besides, functional ability was influenced by age [41, 42] and the older the age, the lower the scores. With the aging process, the older adults would inevitably experience loss of strength, osteoporosis or other degenerative changes, which might increase the risk of diseases and loss of functional ability, and even lead to hospitalization or death. Anyway, age was an important factor that cannot be ignored. The potential explanation of interaction may be that the combined action of multimorbidty status and poor functional ability further increased the likelihood of hospitalization. On one hand, many studies have demonstrated that a greater number of chronic diseases was consistently associated with greater risk of functional dependency. A recent population-based cohort study in Sweden found that the greater dependence of the elderly adults was from multimorbidity [18]. On the other hand, the pattern of positive associations between multimorbidity and hospital resource utilization has been consistently reported across a range of other studies [21, 22, 43]. There is a considerable variation in health care utilization and costs in individuals with and without multiple chronic conditions [44]. Those multimorbid older adults were admitted to hospitals more often than peers without multimorbidity. This agrees with our results that the relevance of FIM domains on hospitalization were found only in the multimorbid group which might be attributed to the existence of multimorbidity- their common risk factor. In summary, the associations of hospitalization and multiple chronic conditions and functional status are complex and not be fully understand and more studies are needed in the future.

We also found that, in the multimorbid group, the independent contribution of the third cluster to hospitalization was even larger than that of socio-demographic characteristics. It might be partially explained by that individuals with multimorbidity are more likely to seek treatment or to be hosptitalized due to the poor disease-related function rather than the individual characteristics. Of the social demographic factors, only gender was significantly associated with annual hospitalization and men had greater odds of hospitalization, as shown in previous studies [9, 10, 45]. However, no association of age on hospitalization was observed in this population, which was likely because age-related risk factors (such as comorbidities) are significant predictors of hospital admissions [46]. Also, multimorbidity is generally related to advanced age, so variation in age was markedly constrained. Additionally, the second cluster had the greatest independent contribution to the hospitalization and the number of chronic diseases was significantly associated with the hospitalization. The unhealthy lifestyle, as risk factors of chronic diseases, and other illnesses could increses the likelihood of the hospitalization. Therefore, the prevention, control and treatment of chronic diseases are essential and urgent.

In the non-multimorbid, age had a significant association with annual hospitalization, similar to a previous study finding that the probability of hospitalization significantly increased with age among older Germans [7]. Our results also showed that some specific diagnosed diseases were found to be significantly associated with hospitalization. Individuals with diabetes mellitus, peripheral vascular disease and heart disease were at greater risk of hospitalization, in accordance with previous studies [5, 10]. Another survey of 18 countries across five regions also observed that inadequate glycemic control and microvascular complications were independent parameters associated with hospitalization [47].

However, this study has several limitations. First, the data in our analyses were based on self-reports, which could lead to biases or tend to inaccuracies. Second, reasons of hospitalization and the length of hospital stay were not included in our analyses. Future studies are needed to provide more detailed information. Additionally, the larger sample size might overestimate certain parameters. Lastly, this is a cross-sectional study, so that the observed associations could not be assumed to be causal relationships. Further in-depth studies with longitudinal follow-up data are warranted to explore the cause-effect relationship.

## Conclusions

A lower FIM score in walk domain was associated with increased risk of annual hospitalization in older Chinese adults with multimorbidity. The findings suggested that there exist some degree of correlation between functional status and annual hospitalization among older adults in Southern China, which might be due to the presence of multimorbidity with advanced age. Tailored interventions for older people may be needed to prevent multimorbidity and improve functional status so as to reduce the risk of hospitalization.

## Abbreviations

ADL: Activities of daily living
BMI: Body mass index
CI: Confidence interval
FIM: Functional Independence Measure
OR: Odds ratio
SD: Standard deviation
